# Structural and Dynamic Features of Liver Mitochondria and Mitophagy in Rats with Hyperthyroidism

**DOI:** 10.3390/ijms232214327

**Published:** 2022-11-18

**Authors:** Natalya Venediktova, Ilya Solomadin, Vlada Starinets, Galina Mironova

**Affiliations:** Laboratory of Mitochondrial Transport, Institute of Theoretical and Experimental Biophysics, Russian Academy of Sciences, 142290 Pushchino, Russia

**Keywords:** mitochondria, energy metabolism, thyroid hormones, mitochondrial dysfunction, quality control system

## Abstract

This work investigated the effect of thyroxine on the biogenesis and quality control system of rat liver mitochondria. Chronic administration of thyroxine to experimental animals induced hyperthyroidism, which was confirmed by a severalfold increase in serum-free triiodothyronine and thyroxine concentrations. The uptake of oxygen was found to increase with a decrease in ADP phosphorylation efficiency and respiratory state ratio. Electron microscopy showed 36% of liver mitochondria to be swollen and approximately 18% to have a lysed matrix with a reduced number of cristae. Frequently encountered multilamellar bodies associated with defective mitochondria were located either at the edge of or inside the organelle. The number, area and perimeter of hyperthyroid rat mitochondria increased. Administration of thyroxine increased mitochondrial biogenesis and the quantity of mitochondrial DNA in liver tissue. Mitochondrial dynamics and mitophagy changed significantly. The data obtained indicate that excess thyroid hormones cause a disturbance of the mitochondrial quality control system and ultimately to the incorporation of potentially toxic material in the mitochondrial pool.

## 1. Introduction

Thyroid hormones (thyroxine (T_4_) and triiodothyronine (T_3_)) play important roles in the metabolism, growth and differentiation of tissues. The mechanisms of action of these hormones have been widely investigated; much remains to be learned, however, about how thyroid hormones (TH) regulate a variety of cellular functions. Excess TH in the blood cause acute intensification of the metabolism and lead to disorders in the functioning of internal organs and systems.

Mitochondria, being dynamic organelles, constantly undergo membrane remodeling through fusion and fission, as well as regulated degradation, mitophagy. These processes contribute to maintaining the functional state of the mitochondrial network and to a rapid response to changes in the energy requirements of the cell. Mitochondrial dynamics include organelle fusion and fission. Such dynamin-related GTPases as mitofusins (Mfn1, Mfn2) and optic atrophy protein 1 (OPA 1) are part of the main mechanism of mitochondrial fusion, the process of combining two or more mitochondria into a single structure. Mitochondrial fission in mammals is mediated by dynamin-related protein1 (Drp1), which is also a large GTPase [1]. Until now, no mechanistic studies have directly correlated TH administration with fusion/fission processes [2]. It is assumed that the fusion of mitochondria enables mixing their contents (including genome copies, proteins, metabolic products), thus providing for mitochondrial DNA (mtDNA) repair and uniform distribution of metabolites [3].

Mitochondrial fission and fusion, as well as motility, play important roles in mitophagy [4]. It refers to a protective effect wherein damaged mitochondria are selectively degraded by lysosomes through autophagic processes in response to various stimuli, including hypoxia, reactive oxygen species (ROS) and respiratory chain inhibitors. It can be initiated both by PINK1 (PTEN induced kinase 1)/PRKN (parkin E3 ubiquitin protein ligase)-dependent and -independent pathways [5]. Several receptors of an independent form of mitophagy have been identified to date: BNIP3 (Bcl-2/adenovirus E1B protein-interacting protein 3), Nix/BNIP3L (Nip3-like protein X), FUNDC1 (FUN14 domain containing 1), cardiolipin [4].

Mitochondrial dynamics, biogenesis and mitophagy are among those that should be clearly regulated by TH [2]. No single molecular model exists to explain the mechanisms of the diverse effects of TH on the organism. Research is carried out on cell lines; on the biological materials of humans, mice, and rats; and by using different concentrations of thyroid hormones or their derivatives as well as their administration techniques. For this reason, no single unambiguously correct answers exist about the effect of TH on mitochondrial metabolism, although significant progress has been achieved over recent years in studying the iodothyronine effects on the respiratory chain. This paper is yet another attempt to determine the mechanism of TH action on the regulation of energy metabolism, mitochondrial quality control and mitophagy in the liver of animals with this model of hyperthyroidism. The obtained results can not only provide new basic knowledge about the functioning of mitochondria but also delineate new prospects for the development of therapeutic strategies for the correction of this pathology and a number of other diseases associated with altered mitochondrial bioenergetics.

## 2. Results

### 2.1. Characterization of Animals with Experimentally Induced Hyperthyroidism

The development of hyperthyroidism was confirmed by measuring the serum concentrations of free T_3_ and T_4_ in rats (Table 1). The levels of T_3_ and T_4_ in hyperthyroid rats (HR) were about 1.8 and 3.4 times higher as compared with the values in control rats (CR). Experimental rats featured a decrease in the liver and body weight as well as in body weight gain (Table 1).

### 2.2. Functioning of Liver Mitochondria of Control and Hyperthyroid Rats

We estimated the functional state of rat liver mitochondria by measuring respiration rates in different metabolic states using NAD-/FAD-dependent substrates and TMPD (nonphysiological electron-donating compound)/ascorbate (Table 2). It was found that the respiration rates of HR liver mitochondria (HLM) in practically all metabolic states (except state_2_,_3_ when using pyruvate/malate) were 20–60% higher than those in CR liver mitochondria (CLM). At the same time, the efficiency of ADP phosphorylation (ADP/O) and respiratory control ratio (RCR) in HLM were decreased.

Our earlier work has shown that the hydrogen peroxide formation and lipid peroxidation rates increase when using this model of hyperthyroidism in liver mitochondria, and the activity of antioxidant enzymes is insufficient to prevent the development of oxidative stress [6]. One of the main elements of antioxidant defense in the cell is the glutathione (GSH) system, which protects the cell from ROS and prevents membrane lipid peroxidation. The present work showed that glutathione metabolism also changed in animals after T_4_ treatment (Table 3).

The concentrations of the total and reduced forms of GSH decreased by about 40% as compared with those in control rats. The redox status (GSH/GSSG), an oxidative stress indicator, in hyperthyroid rats decreased by two times. The concentration of GSSG in liver mitochondria of the investigated animals remained invariable.

### 2.3. Ultrastructural Features of Liver Tissue in Control and Hyperthyroid Rats

**Figure 1:**
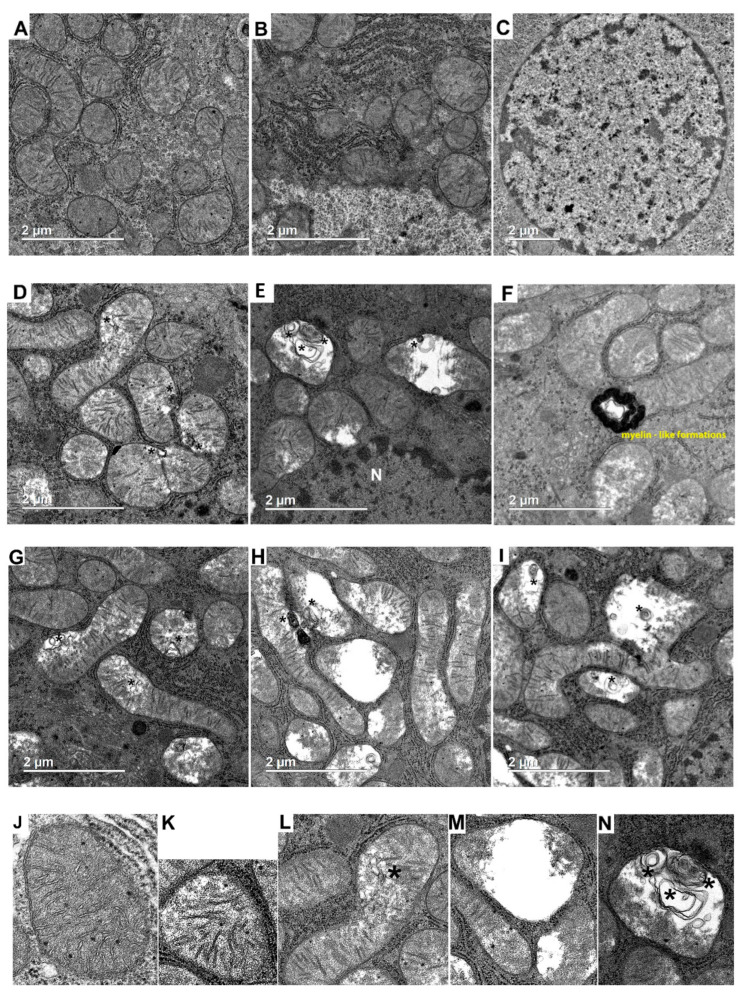

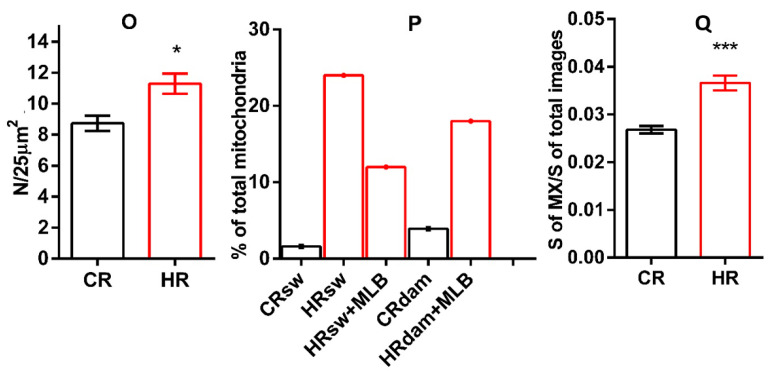
Typical electron micrographs of hepatic tissue in the control (**A**–**C**) and in experimental hyperthyroidism (**D**–**I**). N, nucleus. Scale bar, 2 µm. (**J**–**N**) Electron micrographs of mitochondrial morphological appearance: (**J**)—well-defined, intact, organized membranes and cristae; (**K**)—swollen mitochondria, slightly a decrease in the electron density of the matrix and irregular cristae; (**L**)—swollen mitochondria with MLBs; (**M**)—matrix vacuolization, cristae are almost not defined; (**N**)—damaged mitochondria with MLBs. (**O**–**Q**) Morphometric parameters of liver mitochondria from experimental rats. (**O**) The number of mitochondria per image (25 µm^2^). The number of examined images in each group was about 50. (**P**) Mitochondrial subpopulations occurring in the groups. The number of mitochondria analyzed in each group varied from 500 to 600. (**Q**) The ratio of the mitochondrial area to that of the total image area (25 µm^2^). CR, control; HR, hyperthyroidism; CRsw/HRsw, swollen mitochondria; HRsw + MLBs, swollen mitochondria with MLBs; CRdam, damaged mitochondria; HRdam + MLBs, damaged mitochondria with MLBs. *—multilamellar bodies (MLBs) (**A**–**N**); *—*p* < 0.05 (**O**–**Q**), ***—*p* < 0.001 as compared with the control data (*n* = 2–3 in each group).

Figure 1A–N shows images of the liver tissue of the investigated rats. Liver mitochondria of the control rats are oval or elongated, with well-packed cristae and an electron-dense matrix without disturbances in the position of the cristae (Figure 1A). It can be seen that in the cytoplasm around the mitochondria there is a well-developed rough endoplasmic reticulum studded with numerous ribosomes. The nuclei of CR hepatocytes are of rounded or oval shape, with smooth nuclear envelope (Figure 1A). Evenly distributed chromatin is clearly seen in the nucleus.

In hyperthyroid rats, 36% of mitochondria were in a swollen state (Figure 1D,F,G,K,P). These mitochondria featured a decrease in the electron density of the matrix, but the number of cristae was not significantly altered. In 12% of swollen HLM, we observed multilamellar bodies (MLBs) (Figure 1D,G,L,P). At the same time, many organelles (18%) were damaged (matrix vacuolization), and the number of cristae was reduced. (Figure 1E,H,I,M,P). MLBs located either along the edge or inside the organelle were often associated with these mitochondria (Figure 1E,H,I,N,P). Almost each HR mitochondrion was surrounded by tightly packed cisternae of the rough endoplasmic reticulum. Myelin-like formations were also registered in HR liver mitochondria (Figure 1F). The nucleus featured chromatin condensation and a configuration change of the nuclear membrane (Figure 1E). Figure 1J–N shows the morphological profile of mitochondria found in the liver tissue of the studied rats.

**Figure 2:**
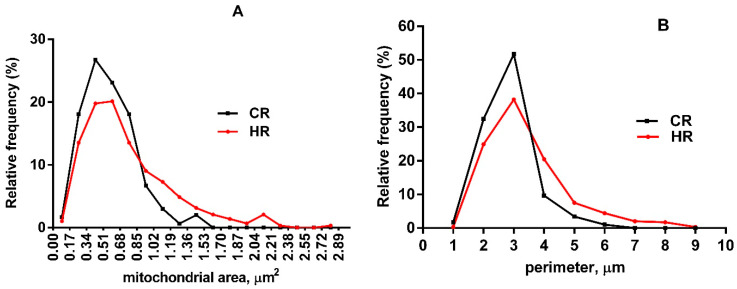
Morphometric parameters of liver mitochondria from experimental rats. (**A**) A histogram of distribution of the mitochondrial area in the groups. (**B**) A histogram of distribution of the mitochondrial perimeter in the groups. The number of mitochondria analyzed in each group varied from 500 to 600. CR, control; HR, hyperthyroidism. (*n* = 3 in each group).

The morphometric analysis of hepatocyte electronic images showed that the number of mitochondria in hyperthyroid rats increased by 23% (Figure 1O). At the same time, the number of damaged mitochondria (matrix vacuolization, membrane/myelin-like formations in organelles) also increased by 3.8 times in HR as compared with CR (Figure 1P). Hyperthyroidism (HT) caused an increase in the area of rat liver mitochondria (Figure 1Q). In HR, we also found mitochondria of an area greater than 1.5 µm^2^ and a perimeter greater than 6 µm (Figure 2A,B). Additionally, the number of mitochondria in an area of 0.3 µm^2^ in HR decreased by almost two times, and the number of mitochondria in a perimeter of 3 µm by 21%.

**Figure 3:**
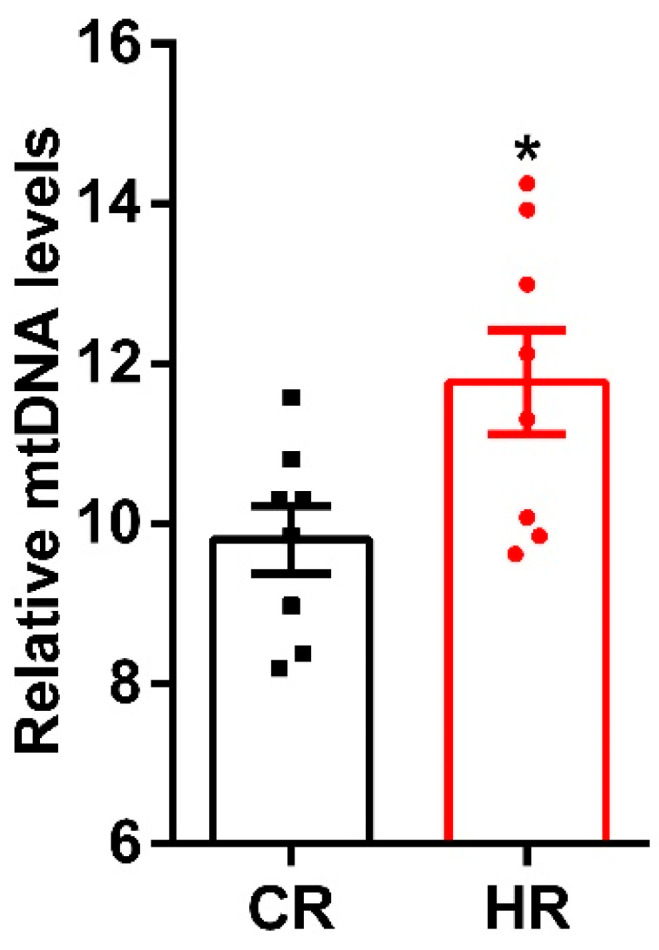
Relative mtDNA levels in the liver of animals. A real-time qPCR was carried out to determine the mtDNA copy number, which is calculated as the ratio mtRNA to nuclear GADPH. CR-control, HR-hyperthyroidism. *—*p* < 0.05 compared with the control data (*n* = 8).

Along with the data on the ultrastructure of liver mitochondria, we assessed the number of mtDNA copies by the PCR. As shown in Figure 3, administration of thyroxine caused a 20% increase in mtDNA levels of HR liver.

### 2.4. Differences of Gene Expression in Liver Tissue of Control and Hyperthyroid Rats

**Figure 4:**
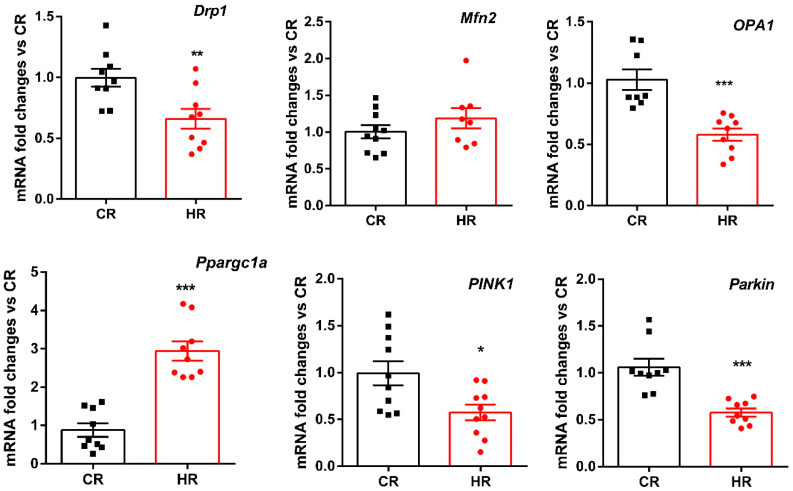
Fold changes of mRNA expression of mitochondrial dynamics, biogenesis and mitophagy genes in liver of experimental animals. CR, control; HR, hyperthyroidism.*—*p* < 0.05, **—*p* < 0.02, ***—*p* < 0.001 as compared with the control data (*n* = 10).

The observed decrease in the efficiency of ADP phosphorylation, as well as the oxidative stress in animals, could be associated with changes in biogenesis and mitochondrial dynamics occurring in the liver during the development of hyperthyroidism. To determine the TH effects on these processes, we measured the expression of genes encoding proteins responsible for mitochondrial fusion (Mfn2 and Opa1) and fission (Drp1), mitochondrial biogenesis (Ppargc1a) and mitophagy (PINK1 and Parkin). It turned out that HR had a three-fold increase in the expression level of Ppargc1a encoding the PGC-1 protein, which mediates mitochondrial biogenesis (Figure 4). The levels of mRNA expression of the gene Mfn2 did not change; at the same time, the expression of two other genes (OPA1 and Drp1) involved in mitochondrial dynamics decreased in the HR liver by 1.7 and 1.4 times, respectively. Additionally, T_4_ caused a significant decrease in Pink 1 and Parkin gene expression compared to CR (Figure 4).

### 2.5. Immunoblotting Analysis of Liver Proteins of Control and Hyperthyroid Rats

**Figure 5:**
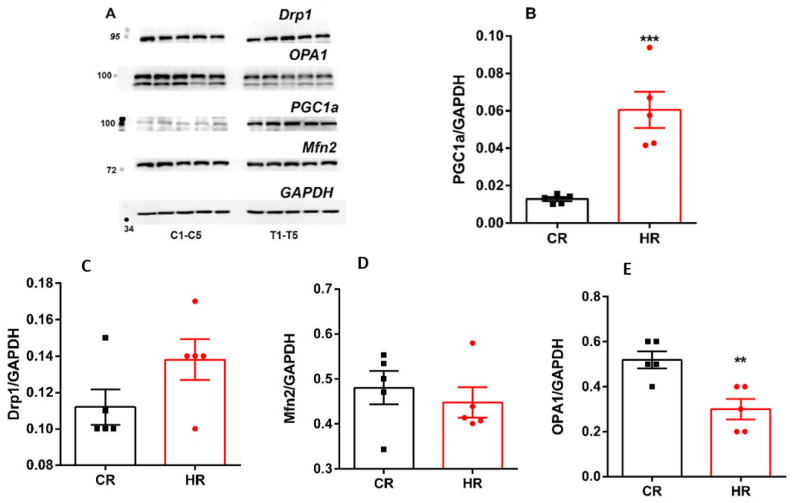
Immunoblotting analysis of mitochondrial dynamics and biogenesis proteins in liver tissue of control and hyperthyroid rats. (**A**) Representative Western blot of Drp1, OPA1, Mfn2, PGC1α, C_1_-C_5_-CR, T_1_-T_5_-HR. (**B**–**E**) Relative levels of appropriate proteins with respect to the loading control (GAPDH). CR, control; HR, hyperthyroidism. **—*p* < 0.02, ***—*p* < 0.001 as compared with the control data (*n* = 5).

The level of proteins responsible for mitochondrial biogenesis, dynamics and mitophagy in investigated rats was analyzed. Significantly increased level of PGC1a was found in the liver of HR (Figure 5). As shown in Figure 5, the quantity of Mfn2 and Drp1 remained unchanged but OPA1 decreased as compared with CR.

**Figure 6:**
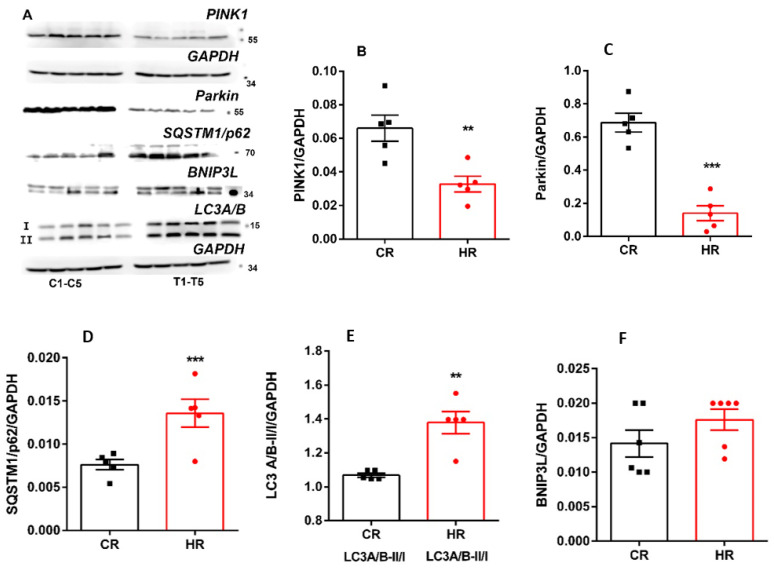
Immunoblotting analysis of mitophagy proteins in liver tissue of control and hyperthyroid rats. (**A**) Representative Western blot of Parkin, PINK1, SQSTM1/p62, LC3A/B-I:II, BNIP3L, C_1_-C_5_-CR, T_1_-T_5_-HR. (**B**–**F**) Relative levels of appropriate proteins with respect to the loading control (GAPDH). CR, control; HR, hyperthyroidism. **—*p* < 0.02, ***—*p* < 0.001 as compared with the control data (*n* = 5–6).

Along with mitochondrial fission and fusion, as well as biogenesis, mitophagy is one of the most important mechanisms for maintaining mitochondrial quality. The most investigated form of mitophagy in mammalian cells is the autophagic pathway mediated by two key proteins: outer mitochondrial membrane kinase PINK1 (PTEN-induced kinase 1) and cytosolic Parkin (E3 ubiquitin ligase). Rats with hyperthyroidism had significant decreases in PINK1 and Parkin levels by two and four times, respectively (Figure 6). We also determined the levels of proteins that are considered autophagy markers, SQSTM1/p62 and LC3A/B (Figure 6). It is believed that elevated levels of SQSTM1/p62 are associated with inhibition of autophagy, while their decreased levels, with activation of the process [7]. It proved that the amount of SQSTM1/p62 increased 1.8 times in HR liver compared with that in CR. The total amount of LC3A/B in HR also increased by 1.9 times. This protein has two isoforms: LC3A/B-I, which is predominantly cytosolic, and LC3A/B-II, which is conjugated with phosphatidylethanolamine. It is the conjugated isoform that occurs in autophagosome and autolysosome membranes and degrades during the autophagy [8]. In this work, the LC3A/B-II/I ratio was increased about 1.3-fold in HR liver compared to CR (Figure 6). Additionally, the level of BNIP3L protein, a receptor involved in a Parkin-independent form of autophagy, was measured. In the state of hyperthyroidism, the amount of BNIP3L in HR did not change (Figure 6).

## 3. Discussion

Thyroid hormones are one of the most important regulators of physiological processes such as metabolism, growth and development in most mammals. Considering the specific action of TH on energy metabolism and the fact that mitochondria are the main component of the cell where metabolic transformations take place makes these organelles a key object of research. In this work, the state of hyperthyroidism was modeled by intraperitoneal administration of thyroxine to animals. Assaying the concentrations of primary diagnostic markers of thyroid activity, T_3_ and T_4_, showed their severalfold increase in blood plasma, which indicated the development of hyperthyroidism in the investigated animals. In addition, there was a decrease in body weight and body weight gain in experimental animals, which is also a characteristic feature of hyperthyroidism (Table 1).

HR liver mitochondria featured an activation of respiration in various metabolic states with a decrease in the efficiency of oxidative phosphorylation and respiratory control ratio (Table 2). Enhanced oxygen consumption can occur under the influence of TH on the activity and/or level of mitochondrial electron transport chain complexes. Indeed, we have experimentally shown an increase in the activity of CI and CII complexes and the quantity of CI, CII and CIV subunits in HR liver mitochondria, but the activity of CII + CIII, CIII and CIV in this case decreases [6]. As for the metabolic inefficiency of oxidative phosphorylation in hyperthyroidism, there are two mechanisms explaining its molecular basis: an effect on the uncoupling of respiration (proton leakage) and an effect on redox proton pumps, the so-called “redox slippage” [2]. Thus, one of the reasons for the decrease in the efficiency of oxidative phosphorylation of ADP in this case can be a malfunction of some electron transport chain (ETC) complexes, leading to a decrease in proton release through the membrane.

Adaptation of mitochondria to various physiological conditions largely depends on the regulation of mitochondrial ultrastructure, especially on the level of cristae [9]. Cristae are the basis for forming oxidative phosphorylation system complexes and assembling respiratory supercomplexes (RSCs) [10]. Such a supramolecular organization presumably has several significant biological features, among which is the decreased formation of ROS [11], the enhanced catalytic function of individual complexes [12] and the increased efficiency of electron transport through channeling [13].

Ultrastructural examination of liver tissue revealed noticeable changes in the mitochondrial population and cell nuclei (Figure 1). Part of the organelles were swollen, with an expanded intercristal space. The number of cristae in these mitochondria did not visually change much (Figure 1D,F,G,K,P). Changes in mitochondrial volume are also found in other HT models. [14,15]. Such mitochondria possibly have an enhanced ability to phosphorylate due to an increase in their surface area and the formation of additional folds on the inner membrane during the swelling [16]. The number, area and perimeter of the HLM increased (Figure 1O,Q and Figure 2A,B). In HR hepatocytes, several megamitochondria were found (of an area greater than 1.5 µm^2^) (Figure 1G–I). Similar data were obtained in HT and other studies [15,17,18]. However, HR also had mitochondria with lysed matrix (18% of the total HLM population), and the cristae in these organelles were reduced in number and shifted to the center or periphery (Figure 1E,H,I,M,P).

These mitochondria were found to have multilamellar bodies [19,20] located either along the edge or inside the organelles (Figure 1E,H,I,N,P). It is possible that at the first stage of HT development the formation of MLBs is associated with an attempt to compensate for the metabolic needs of the cell. However, the occurrence here of aberrant, unattached, vesicular cristae suggests that the formation of MLBs (as cristae junctions) depends on and can be impaired by the dose and duration of TH exposure to the cell [16]. The myelin-like formations observed in the liver are usually phospholipids and occur in the cytoplasm of hepatocytes in a free form or in mitochondria (Figure 1E). Hyperthyroidism also affected the degree of chromatin condensation in HR hepatocyte nuclei, indicating changes in processes related to DNA functioning (Figure 1E).

Maintenance of the number of mitochondria in a cell is mediated by mechanisms of mitochondrial biogenesis and degradation. Administration of T_4_ caused activation of mitochondrial biogenesis, which correlated with an increase in mtDNA in HR (Figure 3, Figure 4 and Figure 5) [21,22].

The mitochondrial quality control system in the cell includes the fusion and fission of mitochondria. In the present work, HT caused a significant change in mitochondrial dynamics, which consisted in a decrease of both the expression of the OPA1/Drp1 genes and the level of OPA1 in HR (Figure 4 and Figure 5). OPA1, responsible for mitochondrial fusion, plays an important role in maintaining the structural organization and integrity of the inner mitochondrial membrane. The deficit of OPA1 worsens the assembly of RCS, leading to a decrease in the activity of ETC and oxidative phosphorylation, which we possibly do observe in this work at the action of TH [23].

Any change in mitochondrial dynamics has its consequences; thus, for instance, the division of the mitochondrial network into smaller-size mitochondria or a change in the activity of OPA1 and Mfn precede the process of organelle degradation [24]. To counteract ROS-induced cell damage and death, T_3_ stimulates mitophagy via Beclin1, PINK1/Parkin and microtubule-associated protein LC3 pathways [22,25,26,27]. However, research on mitophagy in this HT model showed serious violations of this process in the liver of HR. We found that the expression of the genes of the canonical form of PINK1 and Parkin mitophagy, and the level of these proteins decreased in HR as compared to these values in CR (Figure 6). The level of autophagy markers p62 and LC3A/B-II increased in HR (Figure 6). It is likely that an excess of TH activates the initiation of the mitophagic process, which consists in the formation of a phagophore (or an insulating membrane), but its further growth and transformation into an autophagosome is inhibited [28]. Determination of the level of BNIP3L, a non-canonical mitophagy receptor, showed that its quantity did not change as compared with CR (Figure 6).

Thus, a decrease in PINK1/Parkin can lead to a disturbance of mitophagy, and diminished levels of OPA1 prevent mitochondrial fusion processes for content exchange, leading to an impaired homogeneity of the mitochondrial population and accumulation of dysfunctional organelles.

## 4. Materials and Methods

### 4.1. Animals

The research was conducted on 3–3.5-month-old Wistar male rats (weight, 210–230 g). The animals were randomly divided into control and experimental groups (*n* ≥ 5 for each group). Hyperthyroidism was induced by intraperitoneal injection of L-thyroxine at a dose of 200 μg per 100 g of animal weight for 7 days. The control rats were injected with an equal volume of saline. Rats were sacrificed by cervical dislocation at the same time. Plasma levels of free T_3_ and T_4_ were determined by an enzyme-linked assay (Vector-BEST, Russia).

### 4.2. Electron Microscopy

Electron microscopic studies were carried out using pieces of liver taken immediately after decapitation of animals. The tissue was fixed in a 2.5% glutaraldehyde solution in 0.1 M phosphate-buffered saline (PBS, pH 7.4) for 2–3 h, followed by a 2 h incubation in a 1% solution of osmic acid. Subsequently, the samples were dehydrated in increasing concentrations of alcohol and in absolute acetone, and encapsulated in Epon 812 epoxy resin. Ultrathin (60–75 nm) sections were prepared on a Leica EM UC6 microtome (Leica microsystems, Wetzlar, Germany) and stained with uranyl acetate and lead citrate. The preparations were examined and photographed by a Tecnai Osiris FEI (USA). The morphometric analysis of images was carried out using ImageJ 1.52n software; 35–50 electron micrographs for each individual animal (*n* = 2–3) were studied. The different stages of mitochondrial damage are presented: Score 1: healthy mitochondria (well-defined, intact, organized membranes and cristae); Score 2: swollen mitochondria, a slight decrease in the electron density of the matrix and irregular cristae; Score 3: swollen mitochondria with MLBs; Score 4: matrix vacuolization, cristae are almost not defined; Score 5: damaged mitochondria with vacuolized matrix and MLBs.

### 4.3. Quantification of Mitochondrial DNA

Total DNA (nuclear and mtDNA) was extracted from 10 mg deep-frozen liver tissue using the DNA-Extran 2 kit (Sintol, Moscow, Russia) in accordance with the manufacturer’s protocol. The mtDNA level in the liver tissue was evaluated by the PCR [29] and expressed as mtDNA/nuclear DNA (nDNA) ratio. Primers for mtDNA and nDNA are presented in Table 4. The real-time PCR was performed with a QuantStudio 1 Real-Time PCR System (Thermo Fisher Scientific, Waltham, MA, USA) using the qPCRmix-HS SYBR reaction mixture (Evrogen, Moscow, Russia), containing a commonly used fluorescent DNA binding dye SYBR Green II. The comparative C_T_ method was used to quantify the results [30].

### 4.4. Quantification of mRNA Expression of Mitochondrial Dynamics, Mitochondrial Biogenesis and Mitophagy Genes Using the Quantitative Real-Time PCR

The level of gene expression was determined by reverse-transcription real-time PCR. Total RNA from liver samples of control and hyperthyroid rats (about 100 mg) was obtained using ExtractRNA reagent (Evrogen, Moscow, Russia) in accordance with the manufacturer’s protocol. The resulting RNA preparation was treated with RNase-free DNase I (ThermoFisher Scientific, Waltham, MA, USA). The total RNA concentration was measured spectrophotometrically using NanoDrop 1000 (Thermo Scientific, Wilmington, NC, USA). For reverse transcription, we used 2 μg total RNA, MMLV reverse transcriptase (Evrogen, Moscow, Russia) and standard dT15 oligonucleotide (Evrogen, Moscow, Russia). cDNA synthesis was carried out according to the manufacturer’s recommendations (Evrogen, Moscow, Russia). The resulting cDNA was used for the PCR with oligonucleotides specific for the investigated genes. Selection and analysis of gene-specific primers was carried out using Primer-BLAST [31] (the oligonucleotide sequences are presented in Table 5). Real-time PCR was performed by the QuantStudio 1 amplifier (ThermoFisher Scientific, Waltham, MA, USA) using the qPCRmix-HS SYBR kit (Evrogen, Moscow, Russia) with SYBR Green II as a fluorescent intercalating dye. The values of the threshold cycle Ct were determined using the software of the abovementioned instrument. The relative level of expression of each gene was normalized by the level of Actb mRNA; the results obtained were quantified by the comparative CT method. The ∆∆Ct was calculated by the formula ∆∆Ct = ∆Ct (experiment) − ∆Ct (control); each value of ∆Ct was calculated as ∆Ct = Ct (investigated gene) − Ct (housekeeping gene) [30].

### 4.5. Isolation of Rat Liver Mitochondria and Determination of Respiration and Oxidative Phosphorylation

Mitochondria were isolated from the liver by differential centrifugation as described earlier [6]. The isolation medium contained 210 mM mannitol, 70 mM sucrose, 10 mM HEPES-KOH, 0.1% fatty acid-free BSA, 0.5 mM EGTA (pH 7.4). The concentration of the protein was determined by the Lowry method [32].

The rate of oxygen consumption was measured polarographically using Oxygraph-2k (Oxygraph-2k, OROBOROS Instruments, Innsbruck, Austria) at 26 °C under continuous stirring. The reaction medium contained 100 mM sucrose, 50 mM mannitol, 60 mM KCl, 10 mM HEPES, 0.5 mM EGTA-K, 2.5 mM KH_2_PO_4_ (pH 7.4). The concentrations of substrates and other reagents were as follows: 5 mM potassium glutamate, 2.5 mM potassium malate, 5 mM pyruvate, 5 mM potassium succinate, 2 mM ascorbate, 0.5 mM TMPD, 0.2 mM ADP, 50 µM 2,4-dinitrophenol (DNP) [33]. Energetic state definitions: state_2_, basal substrate respiration; state_3_, respiration stimulated by addition of ADP; state_4_, metabolic state upon depletion of all ADP; state_DNP_ (state of uncoupled respiration), respiration in the presence of the uncoupling agent DNP. The RCR (state_3_/state_4_), respiratory states, and ADP/O ratios were determined according to Chance and Williams [34]. The rates of oxygen consumption by mitochondria were expressed as nmol O_2_/min·mg. The concentration of mitochondrial protein in the cuvette was 0.3 mg/mL in the case of TMPD + asc; 0.5 mg/mL, in the case of succ + glu; and 1 mg/mL, in the case of glu + mal/pyr + mal.

### 4.6. Glutathione Assay

GSH was determined by the method based on oxidation of glutathione by the sulfhydryl reagent 5,5-dithio-bis(2-nitrobenzoic acid) (DTNB) to form the yellow derivative 50-thio-2-nitrobenzoic acid (TNB). The rate of TNB formation measured at 412 nm was proportional to the sum of GSH and GSSG present [33].

### 4.7. Immunoblotting Analysis

Total protein extracts were prepared from 10–15 mg of the deep-frozen liver tissue. To maintain extract integrity and function, use was made of Complete Protease Inhibitor Cocktail (P8340, Sigma-Aldrich, St. Louis, MO, USA), Phosphatase Inhibitor Cocktail II (ab201113, UK), PMSF (1 mM), EGTA (1 mM) and EDTA (1 mM). Proteins were isolated using a 1X RIPA buffer (ab156034, USA). The concentration of the protein was determined by the Lowry method [32]. The samples (30–50 µg) were diluted in Laemmli buffer, separated by 12.5% SDS-PAGE and transferred to a 0.45 µm nitrocellulose membrane (Thermo Scientific, Wilmington, NC, USA). Then the membrane was incubated for 1 h with a blocking buffer (5% dry milk dissolved in a PBST buffer) and after that with the appropriate primary antibody overnight at 4 °C. The membranes were washed with a PBST buffer three times at 5 min intervals and were then incubated for 1 h with the appropriate secondary antibody conjugated to horseradish peroxidase (7074, Cell Signaling Technology Inc., Danvers, MA, USA). The peroxidase activity was detected with ECL chemiluminescence reagents (Bio-Rad Laboratories, Hercules, CA, USA). The relative levels of the detected proteins were visualized using a C-DiGit Blot Scanner (LI-COR Biotechnology, Lincoln, NE, USA) and normalized to the appropriate loading control. Optical density measurements were performed using LI-COR Image Studio software. The antibodies used were as follows: (ab56788) anti-DRP1 (1:1000), (ab119685) anti-OPA (1:1000), (ab124773) anti-Mitofusin2 (1:1000), (ab54481) anti-PGC1a (1:1000), (cs2132) anti-Parkin (1:1000), (Affinity Biosciences, DF7742) anti-PINK1 (1:250), (Affinity Biosciences, AF5384) anti-SQSTM1/p62 (1:250), (Affinity Biosciences, DF8163) anti-BNIP3L (1:250), (cs12741) anti-LC3A/B (1:1500). The anti-GAPDH (ab181602) antibodies were used as a loading control.

### 4.8. Statistical Analysis

The data were analyzed using the Graph Pad Prism 6 and Excel software and presented as means ± standard error of the mean (SEM) of 5–10 independent experiments in each group. Statistical differences between the data were determined by the two-tailed *t*-test.

## 5. Conclusions

The data of this study show TH to have an effect on many physiological processes in mitochondria. Excess T_3_ increased oxygen uptake by cells with some decrease in the efficiency of ADP phosphorylation. In addition, a significant increase in biogenesis and imbalance in the content of proteins responsible for organelle fusion and fission in this HT model were noted. The mitophagy process was disrupted in HR, which, together with a decrease in fusion protein (OPA1), probably caused the accumulation of damaged mitochondria, which in turn may have contributed to cell death, the release of pro-apoptotic molecules and/or the formation of increased concentrations of ROS.

## Figures and Tables

**Table 1:** T_3 (free)_ and T_4 (free)_ concentrations, body and liver weights in control and hyperthyroid rats.

	CR	HR
T_3 free_, pmol/L	5.2 ± 0.1	9.3 ± 1.2 ***
T_4 free_, pmol/L	19.2 ± 1.0	66.2 ± 4.4 ***
Body weight, g	256 ± 3.8	236 ± 3.6 **
Body weight gain, g	36 ± 2.6	15 ± 2 ***
Liver weight, g	12 ± 0.3	9 ± 0.2 ***

CR, control; HR, hyperthyroidism. **—*p* < 0.002; ***—*p* ˂ 0.001 as compared with the control data (*n* ≥ 25 in each group).

**Table 2:** The parameters of respiration and oxidative phosphorylation of liver mitochondria of control and hyperthyroid rats.

	Succ + Glu		Glu + Mal		Pyr + Mal		Asc + TMPD	
	CR	HR	CR	HR	CR	HR	CR	HR
State_2_	10 ± 0.4	15 ± 0.6 **	3.2 ± 0.2	4.4 ± 0.2 ***	2 ± 0.2	2.1 ± 0.3	65 ± 6	86 ± 4.8 *
State_3_	71 ± 2.8	95 ± 5 **	43 ± 1.7	56 ± 2.5 ***	20 ± 0.6	18 ± 0.7 *	100 ± 8	127 ± 8 *
State_4_	12 ± 0.4	19 ± 0.6 ***	5 ± 0.3	8 ± 0.2 ***	3.8 ± 0.3	5 ± 0.3 *	63 ± 5	85 ± 4 **
State_DNP_	82 ± 4.2	98 ± 6 *	48 ± 2.3	60 ± 2.5 **	17 ± 0.3	18 ± 0.8	120 ± 11	158 ± 10 *
RCR	5.6 ± 0.1	4.9 ± 0.1 ***	8.2 ± 0.3	7.2 ± 0.3 *	5.6 ± 0.2	3.6 ± 0.2 ***	1.6 ± 0.03	1.5 ± 0.03 *
ADP/O	1.8 ± 0.03	1.7 ± 0.03 **	2.7 ± 0.04	2.4 ± 0.06 ***	2.6 ± 0.1	2.4 ± 0.1 *	0.90 ± 0.05	0.75 ± 0.03 *

State _2_, _3_, _4_, _DNP_-nmol O_2_/min·mg of protein, ADP/O-µmol/ng atoms O. Additions: 5 mM succinate/2.5 mM glutamate, 5 mM glutamate/5 mM pyruvate/2.5 mM malate, 2 mM ascorbate/0.5 mM TMPD, 200 µM ADP (100 µM ADP for asc/TMPD), 50 µM DNP. Mitochondrial protein: 0.5 mg/mL in the case of succ + glu and 1 mg/mL in the case of glu + mal and pyr + mal, 0.3 mg/mL in the case of TMPD + asc. CR-control, HR-hyperthyroidism. *—*p* < 0.05, **—*p* < 0.01, ***—*p* < 0.001 compared with the control data (*n* = 15–20 in each group).

**Table 3:** The concentrations of GSH_total_, GSH_red_, GSSG and the GSH/GSSG ratio in liver mitochondria from control and hyperthyroid rats.

	CR	HR
GSH_total_, nmol/mg	4.9 ± 0.4	3 ± 0.4 **
GSH_red_, nmol/mg	4.8 ± 0.4	2.9 ± 0.04 **
GSSG, nmol/mg	0.13 ± 0.02	0.16 ± 0.03
GSH/GSSG	40 ± 4.4	19 ± 3.1 **

CR, control; HR, hyperthyroidism. **—*p* < 0.02 compared with the control data (*n* = 10 in each group).

**Table 4:** List of the gene-specific primers for the real-time PCR analysis.

Gene	Forward (5′ → 3′)	Reverse (5′ → 3′)
mt-tRNA	AATGGTTCGTTTGTTCAACGATT	AGAAACCGACCTGGATTGCTC
GAPDH	TGGCCTCCAAGGAGTAAGAAAC	GGCTCTCTCCTTGCTCTCAGTATC

**Table 5:** List of gene-specific primers for the real-time PCR analysis.

Gene	Forward (5′ → 3′)	Reverse (5′ → 3′)
Drp1	GATCCAGATGGGCGCAGAAC	ATGTCCAGTTGGCTCCTGTT
Mfn2	AGCGTCCTCTCCCTCTGACA	TTCCACACCACTCCTCCGAC
OPA1	GCAGAAGACAGCTTGAGGGT	TGCGTCCCACTGTTGCTTAT
PINK1	GATGTGGAATATCTCGGCAGGA	TGTTTGCTGAACCCAAGGCT
Parkin	GGCCAGAGGAAAGTCACCTG	CACCCGGTATGCCTGAGAAG
Ppargc1α	TGACATAGAGTGTGCTGCCC	GCTGTCTGTGTCCAGGTCAT
Actb	GACCCAGATCATGTTTGAGACCT	CCAGAGGCATACAGGGACAAC

## Data Availability

The data presented in this study are available on request from the corresponding authors.
